# Paediatric chronic hepatitis C and the global elimination gap: burden and trends from 1990 to 2023 in the Global Burden of Disease Study 2023

**DOI:** 10.1016/j.eclinm.2026.104281

**Published:** 2026-10-24

**Authors:** Suk-Jin Hong, Xinlian Zhang, Farihah Malik, Lynette Goh Suk-Hui, Mortada H.F. El-Shabrawi, Mirta Ciocca, Giuseppe Indolfi, Winita Hardikar, Deirdre Kelly, Kathleen B. Schwarz, Phillipp Hartmann

**Affiliations:** a Department of Pediatrics, University of California San Diego, La Jolla, CA, USA; b Department of Pediatrics, School of Medicine, Daegu Catholic University, Daegu, South Korea; c Herbert Wertheim School of Public Health and Human Longevity Science, University of California San Diego, La Jolla, CA, USA; d University Institute of Public Health, The University of Lahore, Lahore, Pakistan; e Department of Gastroenterology, Hepatology and Nutrition, KK Women’s and Children’s Hospital, Singapore, Singapore; f Department of Paediatrics, Faculty of Medicine, Cairo University, Cairo, Egypt; g Division of Pediatric Hepatology and Transplant Hepatology, Hospital Alemán de Buenos Aires, Buenos Aires, Argentina; h Department Neurofarba, University of Florence, Florence, Italy; i Meyer Children’s Hospital IRCCS, Firenze, Italy; j Gastroenterology and Clinical Nutrition, The Royal Children’s Hospital Melbourne, Parkville, Australia; k University of Birmingham, UK; l Division of Gastroenterology, Hepatology & Nutrition, Rady Children’s Hospital San Diego, San Diego, CA, USA

**Keywords:** Hepatitis C, Global Burden of Disease, Children, Adolescents

## Abstract

**Background:**

Paediatric chronic hepatitis C (PCHC) is often asymptomatic and underdiagnosed, and its contemporary global burden remains incompletely characterised. We estimated global, national, and socioeconomic trends in PCHC using Global Burden of Disease (GBD) 2023 estimates, and hepatitis C virus (HCV)-related liver cancer as a limited Supplementary analysis.

**Methods:**

We analysed GBD 2023 estimates of prevalence, mortality, and disability-adjusted life years (DALYs) for PCHC in people younger than 20 years from 1990 to 2023. Results were summarised globally, across seven official GBD super-regions, and for 204 countries and territories using annual point estimates and supplied 95% uncertainty intervals. Because HCV-related liver cancer estimates were unavailable for children younger than 10 years, analyses of this endpoint were restricted to ages 10–19 years.

**Findings:**

Global PCHC prevalence was 0.380% (95% uncertainty interval [UI] 0.314–0.452) in 2023, representing a decline from 0.443% (0.376–0.514) in 1990, with an absolute change of −0.062 percentage points and a relative change of −14.1%. Mortality declined from 0.0890 (0.0504–0.1538) to 0.0595 (0.0303–0.1023) deaths per 100,000 population younger than 20 years, and the DALY rate declined from 7.49 (4.36–12.92) to 5.06 (2.62–8.40) per 100,000. The highest prevalences clustered in Central Asia, South-East Asia, and sub-Saharan Africa.

**Interpretation:**

The PCHC burden has declined globally, particularly mortality and DALYs, but prevalence has fallen only modestly. The continued high burden in Central Asia, South-East Asia, and sub-Saharan Africa, and marked country heterogeneity highlight the need to integrate antenatal screening, follow-up of perinatally exposed infants, and access to curative treatment for children aged 3 years or older into hepatitis elimination programmes in order to reach the 2030 elimination targets.

**Funding:**

National Institutes of Health.

**Research in context**

**Evidence before this study**

We searched PubMed for articles published from Jan 1, 1989, to May 15, 2026, using combinations of terms for hepatitis C virus, chronic hepatitis C, liver cancer or hepatocellular carcinoma, children, adolescents, paediatric populations, burden, epidemiology, prevalence, mortality, trends, and the Global Burden of Disease. We identified systematic reviews and meta-analyses of paediatric hepatitis C virus (HCV) seroprevalence, a global modelling study estimating that 3.26 million children and adolescents aged 0–18 years were living with chronic viraemic HCV infection in 2018, studies of HCV in pregnancy and vertical transmission, reviews of paediatric HCV treatment, analyses of national HCV policies, and recent cost-effectiveness studies of paediatric direct-acting antiviral (DAA) treatment and childhood screening. These studies established that paediatric HCV is underdiagnosed and often absent from national elimination plans. However, previous evidence was limited by incomplete country coverage, heterogeneous age definitions, reliance on antibody seroprevalence, or absence of comprehensive analyses of long-term trends, mortality, disability-adjusted life years (DALYs), sex-specific patterns, and socioeconomic gradients.

**Added value of this study**

Using GBD 2023, this study provides a global, GBD super-region, and national assessment of paediatric chronic hepatitis C (PCHC) prevalence, mortality, and DALYs from 1990 to 2023, together with an analysis of limited HCV-related liver cancer estimates for children and adolescents aged 10–19 years. We show that PCHC prevalence declined modestly, whereas mortality and DALYs declined more substantially; that prevalence increased stepwise with age; and that residual burden remained concentrated in Central Asia, South-East Asia, and sub-Saharan Africa. Modelled late-period increases after 2018–19 suggest that progress might have slowed, although these signals require cautious interpretation because of incomplete treatment data, possible pandemic-era service disruption, and the rarity of paediatric HCV-related liver cancer.

**Implications of all the available evidence**

The available evidence indicates that paediatric HCV burden is declining but remains a marker of unequal access to screening, maternal–child follow-up, and treatment. Adult-focused elimination campaigns are unlikely to eliminate paediatric HCV unless they are linked to antenatal screening, confirmatory RNA testing, maternal treatment before pregnancy or postpartum according to local guidance, follow-up of perinatally exposed infants, and paediatric DAA access from age 3 years. High-burden settings require targeted support for testing infrastructure, linkage to care, and affordable DAAs.

## Introduction

According to World Health Organization (WHO) estimates, approximately 47 million (95% UI 31–71 million) people were living with a viraemic chronic hepatitis C virus (HCV) infection worldwide in 2024.^1^ Contemporary paediatric estimates are limited. A global modelling study estimated that 3.26 million children and adolescents were living with chronic, viraemic HCV infection in 2018.^2^ Chronic HCV infection is asymptomatic in most infected people, including both children and adults. In children, infection can nevertheless silently progress over many years in an unknown proportion of young people to liver fibrosis and, in some, to cirrhosis. Some children, usually those with advanced fibrotic liver disease may eventually develop hepatocellular carcinoma (HCC).^3^ While severe complications are relatively rare during childhood, and about half of neonates who acquire HCV via maternal–foetal transmission will clear HCV by age 3 years^4^; children who have not cleared the infection by that age usually remain chronically infected into adulthood, contributing to ongoing transmission and increased morbidity and mortality later in life. Moreover, early treatment of paediatric chronic hepatitis C (PCHC) reduces progression to severe liver complications and is cost-effective compared with delaying therapy until adulthood.^5^, ^6^

In 2016, the WHO set a global target to eliminate viral hepatitis by 2030, aiming to reduce new infections by 90% and mortality by 65%.^7^ Achieving this goal requires a comprehensive understanding of disease epidemiology across all age groups, yet data on paediatric HCV remain sparse. In particular, large-scale analyses of global paediatric trends by age, sex, and geographic region, using comprehensive databases such as the Global Burden of Disease (GBD), are limited.

This study aimed to characterise the burden of chronic hepatitis C among children and adolescents using GBD 2023 estimates. We examined global trends and estimates across official GBD super-regions and 204 countries and territories, described age-specific patterns, and explored model-dependent patterns by sociodemographic index, including an HCV-related liver cancer analysis. Our findings will provide valuable insights into the global distribution and long-term trends of PCHC, helping to inform public health strategies aligned with the 2030 elimination goal of the WHO.

## Methods

### Data sources and GBD 2023 methodology

This study is based on data from the GBD Study 2023, coordinated by the Institute for Health Metrics and Evaluation (IHME), to analyse the global epidemiology of PCHC and related liver cancer.^8^ The GBD 2023 study applied a standardised modelling framework that integrates vital registration, surveillance, verbal autopsy, household survey, registry, and published-study inputs to estimate disease burden across 204 countries and territories from 1990 to 2023.^9^ This report is a secondary analysis of publicly accessible aggregate GBD results.

We extracted estimates for prevalence, mortality, and disability-adjusted life years (DALYs), including years of life lost (YLLs) and years lived with disability (YLDs), for individuals under 20 years of age. A DALY represents one year of healthy life lost and is calculated as the sum of YLLs due to premature mortality and YLDs.^9^ In this analysis, DALYs, YLLs, and YLDs attributed to chronic hepatitis C were obtained directly from GBD 2023. The GBD dataset provides data stratified into standardized 5-year age intervals (<5, 5–9, 10–14, and 15–19 years). Global analyses used the aggregate age group younger than 20 years. Age-specific analyses used the four individual age groups. Prevalence rates supplied per 100,000 were divided by 1000 to express population percentages. HCV-related liver cancer was analysed separately among individuals aged 10–19 years because data were not available for children younger than 10 years in GBD 2023. Rates for the official GBD aggregate age group younger than 20 years use the full population aged 0–19 years as the denominator. Results were evaluated globally, across the seven GBD super-regions, and across the 204 countries and territories included in the GBD analysis. Rates were used for temporal and geographic comparisons, while absolute number estimates were used to describe the absolute global burden.

Anti-HCV positivity could indicate either previous exposure or current infection and, therefore, does not by itself establish current infection. GBD estimates of chronic HCV infection are derived from anti-HCV seroprevalence data, which are converted to estimates of current RNA-positive infection using a meta-analytically derived RNA-positive ratio and adjusted for treatment-related cure.^9^ The results are therefore model-derived estimates of chronic HCV infection rather than direct counts of RNA-confirmed children. GBD 2023 estimated chronic hepatitis C using anti-hepatitis C virus seroprevalence inputs within the DisMod-MR 2.1 framework.^10^ DisMod-MR 2.1 is a Bayesian meta-regression framework that synthesises heterogeneous epidemiological inputs across age, sex, location, and year while maintaining consistency among incidence, prevalence, remission, and excess mortality. For chronic HCV, the model incorporated anti-HCV inputs, predictive covariates, age and sex splitting, crosswalk adjustments, conversion to estimated current infection, and treatment adjustments. In the chronic HCV nonfatal model, sociodemographic index (SDI) was used as a predictive covariate.^9^ To account for antiviral treatment, GBD incorporated treatment data from 34 countries assembled with the Coalition for Global Hepatitis Elimination. Where available, treatment inputs were stratified by age, sex, and regimen, including direct-acting antiviral, interferon, and triple-drug regimens.^10^

Where sex-stratified inputs were unavailable, GBD used meta-regression with Bayesian priors, regularisation, and trimming (MR-BRT) to derive modelled female-to-male patterns and split both-sex estimates. Broad age inputs were redistributed into five-year age groups using modelled age patterns and population weights. Non-representative sources such as blood-donor studies were adjusted by modelled crosswalks. These procedures mean that sex- and age-specific contrasts are partly model-dependent and were treated descriptively.^10^

Mortality estimates were obtained through the GBD cause-of-death framework. Cause-specific mortality was modelled with the Cause of Death Ensemble model (CODEm), which evaluates multiple model specifications and covariate sets, assesses predictive performance, and combines better-performing models.^9^ Chronic hepatitis C prevalence, mortality, YLLs, YLDs, and DALYs were reported with supplied 95% uncertainty intervals (UIs).^9^, ^10^ The supplied lower and upper bounds represent the 2.5th and 97.5th percentiles of the GBD uncertainty distribution.

Approval to use GBD data for this publication was obtained from IHME. The analysis used publicly accessible aggregate data and was reported in accordance with the Guidelines for Accurate and Transparent Health Estimates Reporting (GATHER).^11^

### Sociodemographic index

SDI is a composite index ranging from 0 to 1 that incorporates lag-distributed income per capita, mean educational attainment among people aged 15 years and older, and fertility among females younger than 25 years.^9^ Each country or territory was assigned its 2023 SDI value and GBD SDI group. For chronic HCV nonfatal estimation, DisMod-MR 2.1 incorporated SDI as a predictive covariate.^10^ Accordingly, the 2023 SDI displays are descriptive summaries of published estimates rather than independent causal analyses.

### Statistical analysis

The GBD rate metric for prevalence, expressed per 100,000 population, was converted to a population percentage by dividing by 1000. Global annual point estimates and supplied 95% UIs were plotted from 1990 to 2023. Absolute change was calculated as the 2023 point estimate minus the 1990 point estimate, and relative change as ([2023 estimate/1990 estimate] − 1) ∗ 100. Modelled annual estimates were not treated as independent observations. Therefore, temporal correlations and between-group hypothesis tests were not applied. Official aggregate GBD estimates were used for super-region comparisons, and country estimates were presented. SDI displays were considered descriptive and model-dependent because SDI was a predictive covariate in GBD chronic HCV modelling.^9^ No missing GBD estimates were imputed. Analyses and figures were prepared in R version 4.4.1 (R Foundation for Statistical Computing).

### Ethics

The requirement for written informed consent was waived, and formal ethics approval was not required, as the study utilized publicly available, de-sensitized data through IHME that did not contain confidential or identifiable patient information. This study adheres to the Declaration of Helsinki.

### Role of the funding source

The funders of the study had no role in study design, data collection, data analysis, data interpretation, or writing of the report. The authors were independent from the funders.

## Results

### Prevalence of chronic hepatitis C

The global prevalence of PCHC was 0.380% (95% UI 0.314–0.452) in 2023, a decrease from 0.443% (0.376–0.514) in 1990 (Fig. 1A and Supplementary Table S1). This corresponded to an absolute point-estimate change of −0.062 percentage points and a relative change of −14.1%. The annual point estimate declined through the late 2010s and then increased modestly. Of note, despite the lower global prevalence of PCHC in percent, the estimated global absolute number of people younger than 20 years living with chronic hepatitis C increased slightly from 9.98 million (8.49–11.58 million) in 1990 to 10.16 million (8.40–12.08 million) in 2023.

**Fig. 1:**
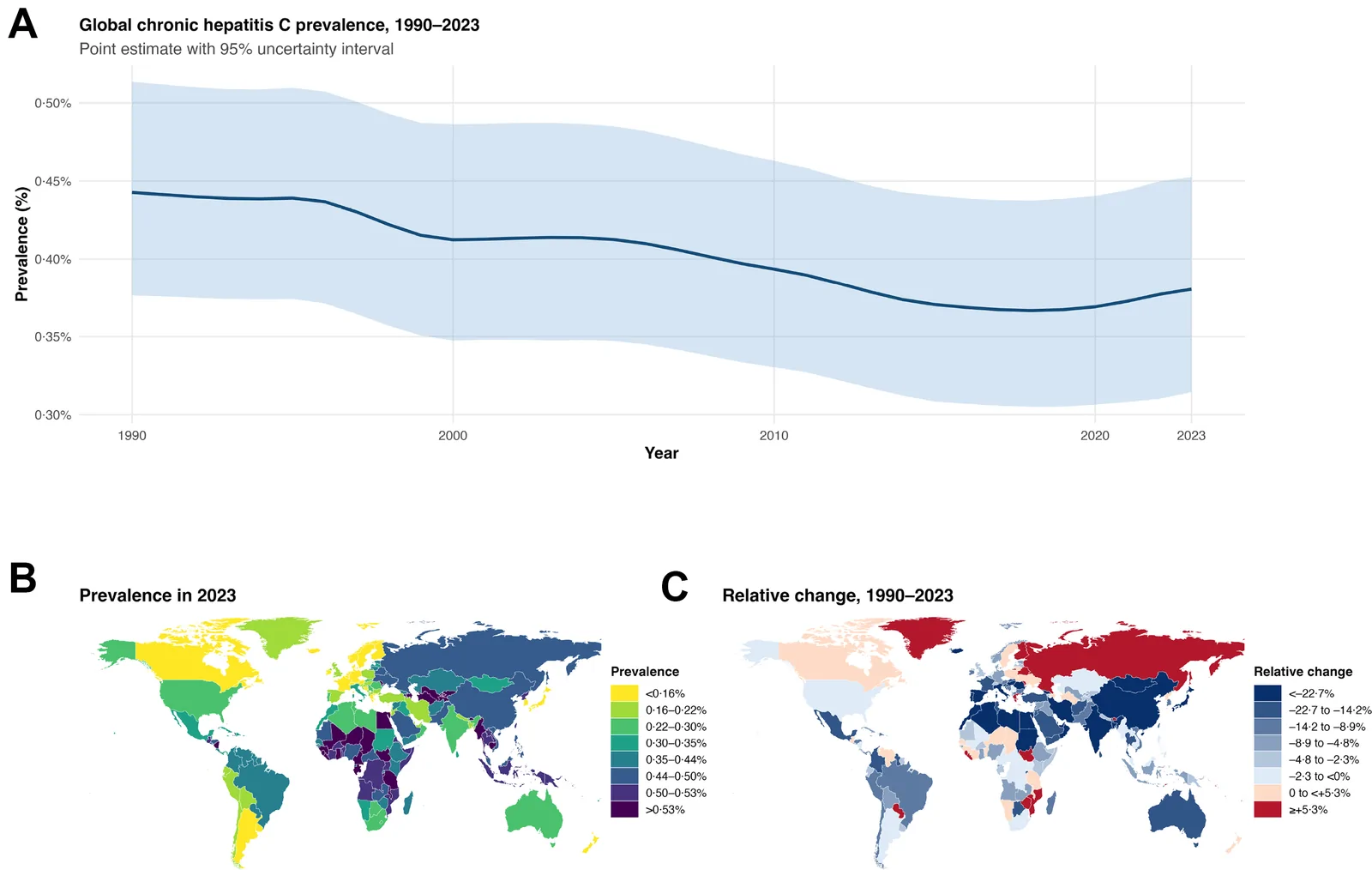
**The prevalence of chronic hepatitis C in children and adolescents.** (A) Global estimates from 1990 to 2023. The solid line indicates the point estimate and the shaded band indicates the 95% uncertainty interval. (B) Country and territory prevalence estimates in 2023. (C) Relative change in the prevalence estimate from 1990 to 2023. Prevalence is expressed as a percentage of the total population younger than 20 years.

Country estimates varied substantially in 2023. The highest prevalence estimates were in Turkmenistan (0.646%, 95% UI 0.555–0.752), Myanmar (0.645%, 95% UI 0.549–0.744), Cambodia (0.633%, 95% UI 0.544–0.733), Egypt (0.595%, 95% UI 0.511–0.679), and Ghana (0.591%, 95% UI 0.510–0.681), with additional high estimates in parts of Central Asia and sub-Saharan Africa. The lowest prevalence was estimated in several high-income European settings, including Iceland (0.088%, 95% UI 0.055–0.126), Norway (0.097%, 95% UI 0.071–0.125), the Netherlands (0.104%, 95% UI 0.081–0.133), and Sweden (0.107%, 95% UI 0.081–0.135) (Fig. 1B and Supplementary Table S2). Most countries showed a decline in prevalence from 1990 to 2023, with the largest relative reduction observed in Mongolia (−54.4%), followed by Georgia (−41.6%) and Spain (−37.6%). In contrast, several countries demonstrated increases in prevalence rate, with the largest relative increases observed in Zimbabwe (+24.3%), American Samoa (+24.2%), and Bhutan (+23.2%) (Fig. 1C).

### Mortality and DALYs of chronic hepatitis C

The global chronic hepatitis C mortality rate was 0.0595 (95% UI 0.0303–0.1023) deaths per 100,000 population younger than 20 years in 2023, a relative point-estimate change of −33.1% from 0.0890 (0.0504–0.1538) deaths per 100,000 in 1990 (Fig. 2A and Supplementary Table S1). In absolute terms at the global level, estimated deaths declined from 2008 (1136–3468) in 1990 to 1590 (810–2732) in 2023. The estimated mortality rates in 2023 were highest in Niger (0.3546 (95% UI 0.1397–0.7149) per 100,000) and other countries in Sub-Saharan Africa, and lowest in European countries. Country mortality estimates and their relative changes are shown in Fig. 2B and C and Supplementary Table S2.

**Fig. 2:**
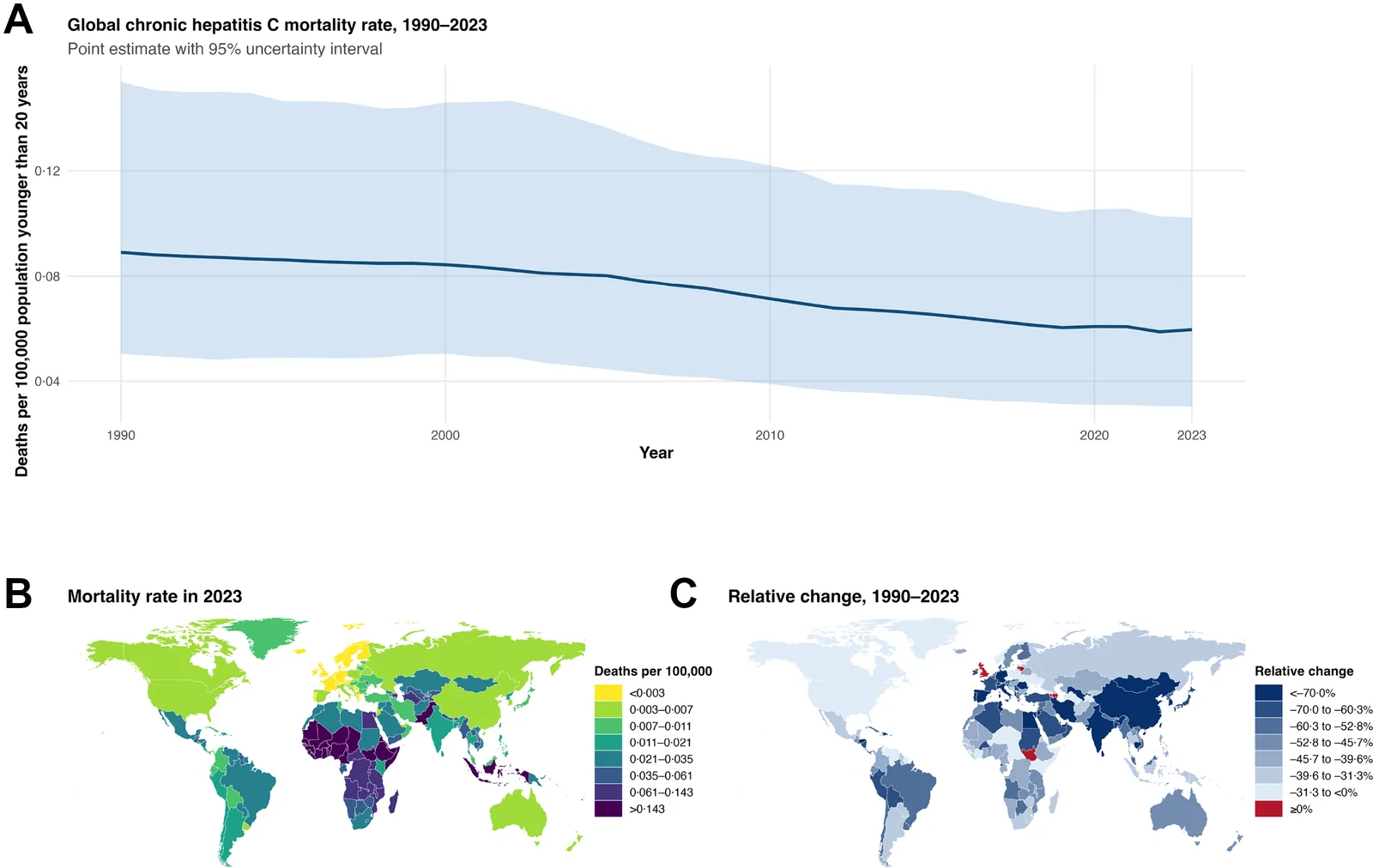
**The mortality of chronic hepatitis C in children and adolescents.** (A) Global mortality rate from 1990 to 2023 with the 95% uncertainty interval. (B) Country and territory mortality rates in 2023. (C) Relative change in the mortality rate estimate from 1990 to 2023. Mortality rates are deaths per 100,000 population younger than 20 years.

The global DALY rate declined from 7.49 (95% UI 4.36–12.92) to 5.06 (2.62–8.40) DALYs per 100,000 population younger than 20 years, a relative point-estimate change of −32.5% (Fig. 3A and Supplementary Table S1). In absolute numbers, estimated DALYs declined from 168,860 (98,334–291,237) to 135,033 (69,839–224,456) globally. Country DALY estimates and relative changes are shown in Fig. 3B and C and Supplementary Table S2. YLLs accounted for most DALYs (Supplementary Table S1).

**Fig. 3:**
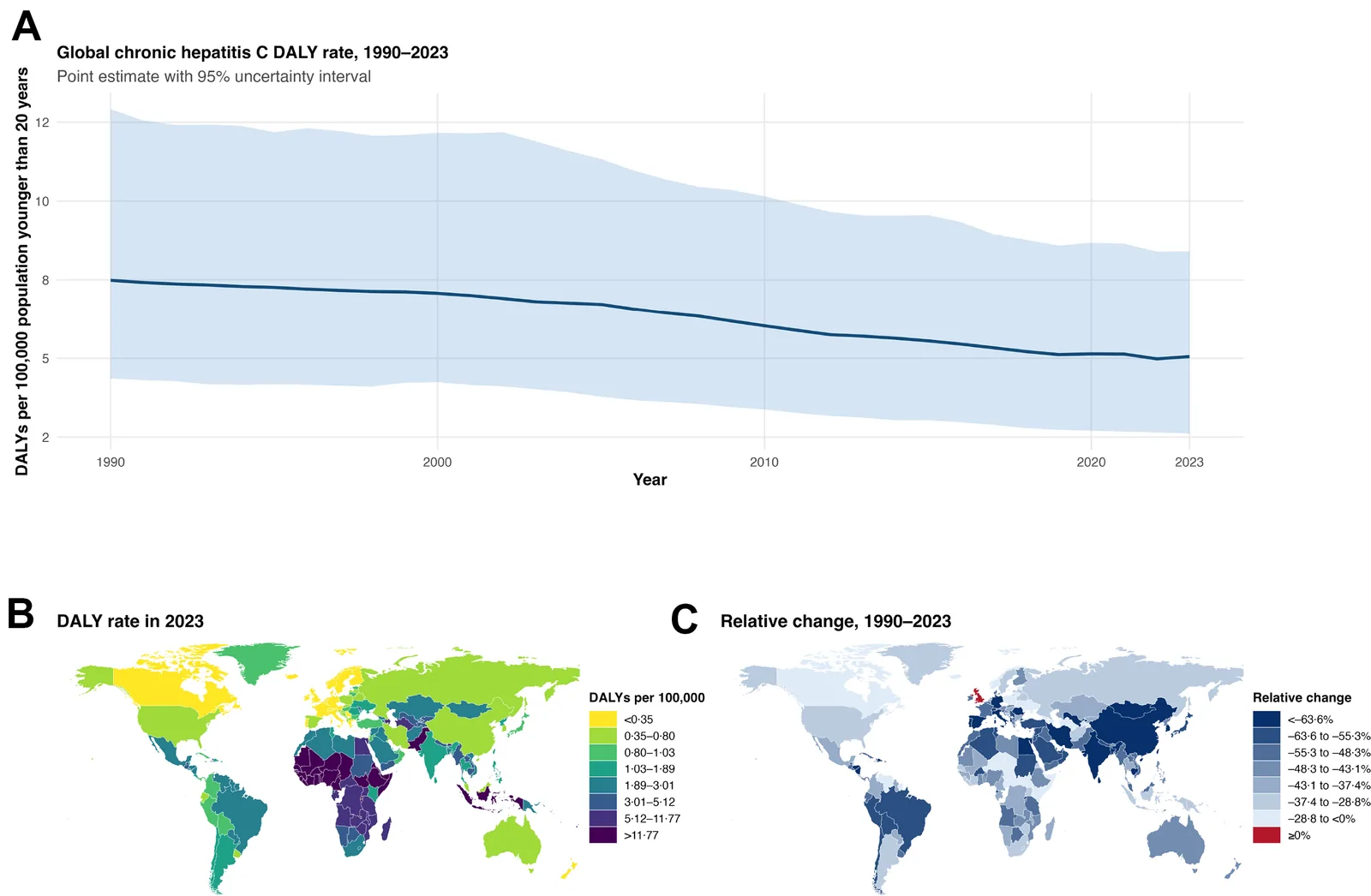
**The disability-adjusted life years (DALYs) of chronic hepatitis C in children and adolescents.** (A) Global DALY rate from 1990 to 2023 with the 95% uncertainty interval. (B) Country and territory DALY rates in 2023. (C) Relative change in the DALY rate estimate from 1990 to 2023. DALY rates are DALYs per 100,000 population younger than 20 years.

Further, most countries demonstrated a decline in mortality and DALYs, with the highest relative reductions observed in Eastern Asia, where South Korea had the largest decrease in both measures from 1990 to 2023 (Supplementary Table S2).

### GBD super-regions

In 2023, chronic hepatitis C prevalence was highest in sub-Saharan Africa at 0.488% (95% UI 0.410–0.571) and lowest in the high-income super-region at 0.181% (0.134–0.235) (Fig. 4 and Supplementary Table S3). Sub-Saharan Africa also had the highest mortality rate at 0.1469 (0.0729–0.2741) deaths per 100,000 and DALY rate at 12.21 (6.15–22.21) per 100,000. The corresponding high-income estimates were 0.0049 (0.0027–0.0082) deaths and 0.45 (0.25–0.75) DALYs per 100,000. Prevalence estimates declined in six of the seven super-regions (excluding Central Europe, Eastern Europe, and Central Asia), while mortality and DALY estimates declined in all seven super-regions from 1990 to 2023.

**Fig. 4:**
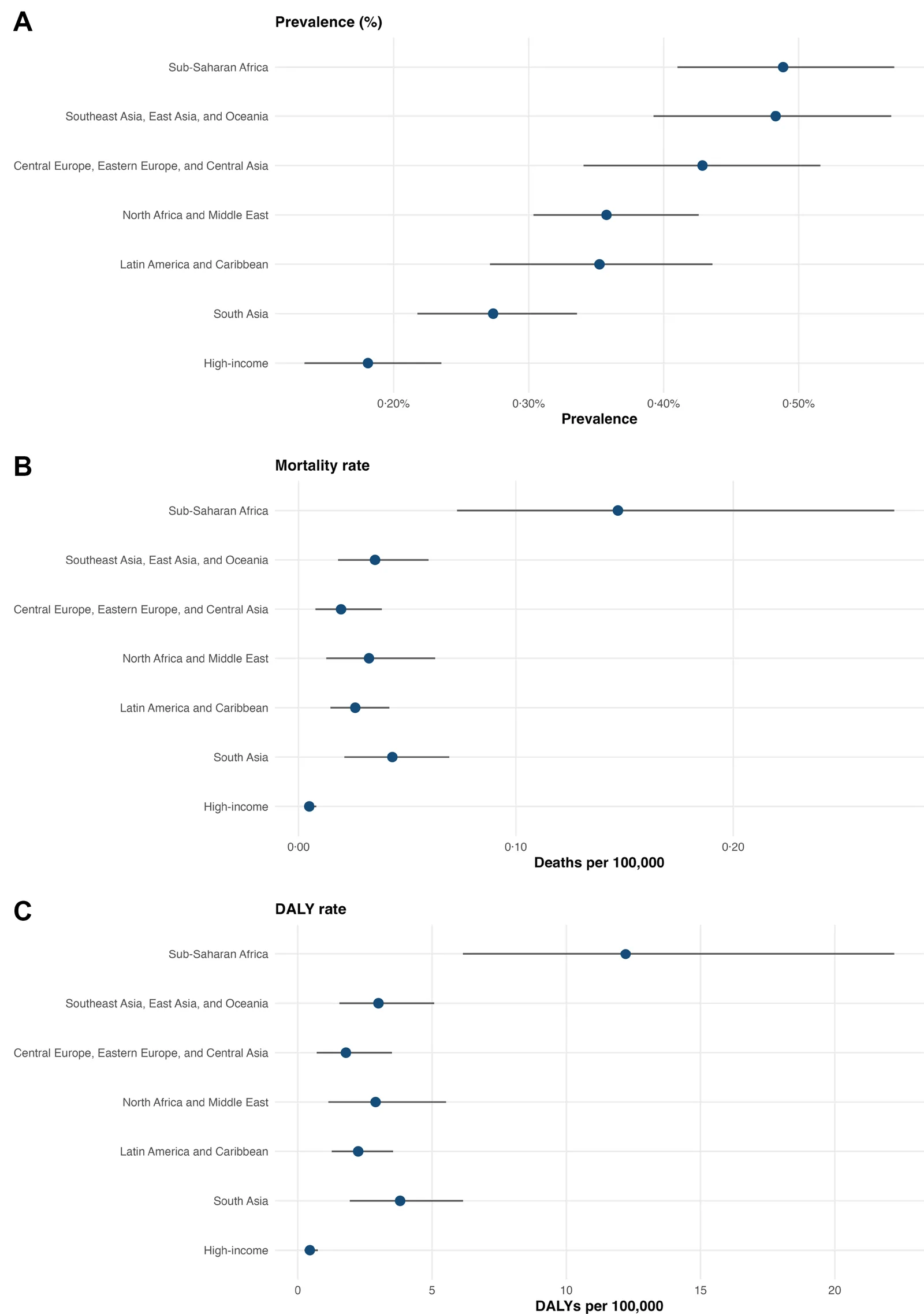
**Chronic hepatitis C burden by GBD super-region in children and adolescents in 2023.** Aggregate GBD estimates for both sexes combined are shown for (A) prevalence, (B) mortality rate, and (C) DALY rate. Points indicate point estimates and horizontal lines indicate 95% uncertainty intervals. Prevalence is a population percentage. Mortality and DALY rates are per 100,000 population younger than 20 years. DALY, disability-adjusted life year; GBD, global burden of disease.

### Prevalence by age group

Chronic hepatitis C prevalence estimates were consistently lowest in the youngest age group and highest in adolescents aged 15–19 years (Supplementary Fig. S1 and Supplementary Table S4). In 2023, prevalence estimates were 0.088% (95% UI 0.069–0.107) among children younger than 5 years, 0.274% (0.222–0.330) at ages 5–9 years, 0.472% (0.388–0.565) at ages 10–14 years, and 0.691% (0.569–0.826) at ages 15–19 years.

### Paediatric HCV-related liver cancer

Paediatric HCV-related liver cancer estimates were small compared with total chronic hepatitis C estimates. In 2023, GBD estimated 63.2 (95% UI 30.3–120.4) prevalent cases, 11.9 (4.8–22.6) deaths, and 926.7 (375.1–1764.9) DALYs globally for ages 10–14 years. At ages 15–19 years, the corresponding estimates were 99.3 (47.9–193.1) prevalent cases, 18.5 (7.8–34.2) deaths, and 1349.8 (569.1–2497.5) DALYs. Global rates, annual trends, and the 2023 prevalence map are shown in Supplementary Fig. S2 and Supplementary Tables S5 and S6. As for chronic hepatitis C, YLL accounted for most DALYs due to HCV-related liver cancer (Supplementary Table S5).

### Chronic hepatitis C by SDI

In 2023, official SDI-group aggregates showed the highest chronic hepatitis C prevalence in the Middle SDI group at 0.454% (95% UI 0.375–0.537) and the lowest in the High SDI group at 0.307% (0.241–0.380). Mortality and DALY rates were highest in the Low SDI group at 0.1173 (0.0589–0.2130) deaths and 9.85 (5.05–17.41) DALYs per 100,000, and lowest in the High SDI group at 0.0081 (0.0045–0.0137) deaths and 0.77 (0.42–1.31) DALYs per 100,000 (Supplementary Table S7). Country estimates across continuous SDI values are shown descriptively in Supplementary Fig. S3.

## Discussion

Using GBD 2023 estimates, we found that the global burden of PCHC has declined since 1990, but the decline was uneven across metrics and across populations. PCHC prevalence decreased modestly, from 0.443% in 1990 to 0.380% in 2023 (relative change of −14.1%), whereas mortality and DALY rates declined by about one-third. Sub-Saharan Africa had the highest super-region estimates for prevalence, mortality, and DALYs in 2023, and country estimates were heterogeneous. Paediatric HCV-related liver cancer continued to be rare.

Our 2023 estimate should be interpreted in the context of earlier paediatric estimates rather than as a directly comparable update as some estimates are based on anti-HCV positivity which indicates exposure to HCV but does not, by itself, distinguish between current and resolved infection, while other estimates are based on HCV RNA. Systematic reviews of paediatric anti-HCV seroprevalence reported higher pooled estimates of 0.87% and 0.904%,^12^, ^13^ whereas Schmelzer et al., ’s 2018 estimates indicate RNA-positive prevalence of 0.13% among children aged 0–18 years,^2^ and Indolfi and colleagues reported a similar estimate of 0.15% HCV RNA prevalence among 1–19-year-olds.^3^ The higher GBD prevalence estimate 0.38% compared with previous viraemia-based paediatric models warrants particular caution. The estimates of the different studies derive from substantially different input data and modelling frameworks. E. g. the CDA analysis by Schmelzer^2^ combined paediatric prevalence data, country-specific disease burden models, age-specific regression models, and regional extrapolation, whereas GBD derives chronic HCV estimates from anti-HCV inputs converted to estimated RNA positivity within DisMod-MR, with treatment adjustment and model-based age and sex allocation where directly stratified data are unavailable. Accordingly, the over two-fold difference should be interpreted primarily as evidence of substantial model uncertainty in the poorly observed global paediatric HCV burden rather than as evidence that paediatric prevalence increased between the two studies. The age gradient also differed substantially. Further, Schmelzer and colleagues^2^ estimated a more modest increase across paediatric age groups, whereas GBD 2023 prevalence increased from 0.088% among children younger than 5 years to 0.691% among adolescents aged 15–19 years. Both models therefore identify increasing prevalence with age, but the substantially steeper GBD gradient is likely sensitive to differences in age-specific input data and modelling assumptions. Because directly observed age-specific paediatric HCV data remain sparse, we regard the direction of this pattern as more robust than its precise magnitude.

Further, the aforementioned higher anti-HCV seroprevalence estimates^12^, ^13^ than viraemia-based estimates^2^, ^3^ are expected, as they include children with past infection and spontaneous clearance. Recent work on pregnancy and vertical transmission provides aetiological context. Quek and colleagues estimated global HCV seroprevalence in pregnancy and mother-to-child transmission,^14^ and Trickey and colleagues estimated annual vertical infections by country and region.^15^ The regional concentration of vertical transmission in these analyses is consistent with the high-burden regions identified in our study, supporting perinatal exposure as a key pathway sustaining paediatric infection.

The burden of PCHC was concentrated in regions where maternal testing, treatment access, and maternal–child health linkage are likely to remain incomplete. In 2023, the highest country-level prevalences clustered in Central Asia, South-East Asia, and sub-Saharan Africa, whereas the lowest estimates were observed in high-income European settings. These gradients indicate that paediatric HCV is not only a virological legacy of past transmission but also a marker of current health-system capacity, including access to screening, confirmatory RNA testing, safe blood and injection practices, maternal diagnosis, and paediatric DAA treatment. The Egyptian experience illustrates this point: large adult screening and treatment campaigns can substantially reduce population-level HCV burden, but residual paediatric infection can persist when antenatal and younger-child pathways are not explicitly included.^16^, ^17^

The modest late-period (post-2019) increase in PCHC burden in GBD 2023 estimates should be interpreted cautiously as these are modelled estimates. However, these signals are relevant because they coincide with documented disruptions to HCV testing and treatment during the COVID-19 pandemic,^18^, ^19^ the delayed incorporation of children into DAA programmes, and incomplete treatment uptake data for many countries during 2020–23. National surveillance in the US recorded 235 perinatal HCV cases in 2023, 38 more than in 2022,^20^ despite decreasing HCV testing of perinatally exposed infants in recent years as noted in some parts of the US.^21^ Similarly, national surveillance data from China showed a 6.13% increase in HCV incidence in adults in 2021 relative to 2020.^22^ Paediatric burden is likely mediated indirectly through reduced maternal diagnosis, delayed linkage to postpartum treatment, incomplete follow-up of perinatally exposed infants, and reduced diagnosis or treatment of adolescents.

The age distribution of PCHC was consistent with a life-course pattern of paediatric HCV infection. In 2023, prevalence increased stepwise from 0.09% in children younger than 5 years to 0.69% in adolescents aged 15–19 years, and this gradient was seen throughout the study period. This finding is consistent with previous paediatric modelling estimates.^2^ However, the lower prevalence in the youngest group likely reflects the combined effects of persistence of infection among children who do not spontaneously clear HCV by early childhood and incomplete ascertainment in settings without routine infant or paediatric testing.^3^, ^4^, ^23^ Horizontal exposure becomes increasingly relevant during adolescence, particularly among young people at risk of acquisition via needle use and sexual transmission, but this route contributes mainly to older paediatric age groups and to specific high-risk subpopulations.^24^ A practical implication is that early childhood and adolescence require different programme responses: the former needs perinatal case-finding, follow-up, and eventual treatment for those 3 years and older; whereas the latter requires opportunities for diagnosis, prevention, and linkage to treatment that are tailored to adolescents.

These findings translate into four strategic priorities. First, national HCV elimination plans should explicitly include children, adolescents, and pregnant women rather than assuming that adult treatment scale-up will indirectly reach paediatric populations.^7^, ^23^ A global policy review found that many national plans lack paediatric testing or treatment recommendations,^23^ and strengthening paediatric prevalence surveillance remains necessary. Second, antenatal screening must be linked to confirmatory RNA testing, maternal treatment before pregnancy or postpartum according to local guidance, and systematic testing of perinatally exposed infants. Studies of pregnancy and vertical transmission show that maternal infection remains a continuing source of paediatric HCV,^14^, ^15^ and CDC guidance recommends HCV RNA testing for all perinatally exposed infants at age 2–6 months^25^; although this approach may be less feasible in low- and middle-income countries where assessing for persistence of anti-HCV antibodies beyond 18 months of age may still be an effective and more affordable strategy. Models from high-burden settings further suggest that integrating antenatal screening with maternal and child treatment pathways could be economically favourable.^17^ Third, paediatric DAA access should be treated as a core elimination activity. Current guidance recommends DAA treatment with an approved regimen for all children and adolescents with HCV infection aged 3 years or older,^26^ and economic analyses support early paediatric treatment and one-time childhood screening strategies in appropriate settings.^5^, ^6^ Fourth, global health stakeholders should prioritise regions where high prevalence intersects with weak screening infrastructure, constrained DAA access, and limited maternal–child follow-up, including parts of Central Asia, Sub-Saharan Africa, and South-East Asia.^1^, ^27^

This study has several limitations. First, all estimates were derived from GBD 2023 and therefore depend on the quality, representativeness, and completeness of the underlying input data. Primary data for paediatric HCV are sparse in many low-income and middle-income settings, and GBD estimates in these countries rely partly on statistical modelling, regional borrowing of information, and imputation, which likely underestimates the real burden.^8^, ^9^, ^10^ Second, chronic HCV prevalence in GBD is derived from anti-HCV seroprevalence, converted to estimated RNA positivity, and adjusted for treatment coverage; residual error is therefore possible when primary serological data are old, non-representative, or not age- and sex-specific.^10^ This issue is particularly relevant in young children because spontaneous clearance after vertical acquisition is common,^4^ and because many national HCV policies still lack explicit paediatric testing or treatment recommendations.^23^ Third, HCV-related liver cancer estimates were very low and not available for children younger than 10 years in GBD 2023.^8^, ^9^ We reported age-specific global counts and rates. Finally, DALYs may underestimate the long-term value of paediatric HCV diagnosis and treatment because GBD assigns little or no disability weight to asymptomatic chronic HCV infection and compensated cirrhosis.^10^ Since most infected children remain asymptomatic for years, paediatric DALYs capture only a small part of the lifetime burden that early DAA treatment may prevent.^5^, ^6^, ^26^

In conclusion, GBD 2023 estimates suggest that the global burden of paediatric chronic hepatitis C has declined over the past three decades, particularly for mortality and DALYs, but that progress has been uneven across different countries and regions. Burden remains concentrated in low-SDI settings and in parts of Central Asia, Sub-Saharan Africa, and South-East Asia, while paediatric HCV burden increases suggest vulnerability in the elimination trajectory. Paediatric HCV elimination will require national programmes to move beyond adult-focused treatment scale-up and explicitly integrate antenatal screening, follow-up of perinatally exposed infants, and DAA access for children and adolescents.

## Contributors

S.J.H. and P.H. directly accessed and verified the underlying data reported in the manuscript and analysed the data. S.J.H. wrote the manuscript. X.Z. provided statistical assistance. S.J.H., X.Z., F.M., L.G.S.H., M.H.F.E.S, M.C., G.I., W.H., D.K., K.B.S., and P.H. interpreted the data and edited the manuscript. P.H. designed the study, supervised the work, and is the guarantor of the study. All authors have read and approved the submitted version.

## Data sharing statement

The raw data is available at https://vizhub.healthdata.org/gbd-results/.

## Editor note

The Lancet Group takes a neutral position with respect to territorial claims in published maps and institutional affiliations.

## Declaration of interests

K.B.S. has a research grant and consults with Gilead Sciences; she is also a consultant for UpToDate. P.H. served on the Hepatology Committee from 2022 to 2025 and currently serves as co-facilitator of the Metabolic Dysfunction-Associated Steatotic Liver Disease (MASLD) and Obesity Special Interest Group of the North American Society for Paediatric Gastroenterology, Hepatology and Nutrition (NASPGHAN). The other authors do not have any competing interests related to this manuscript.

## Appendix A Supplementary data

Supplementary Figs. S1–S3 **Figure S1. Global age-specific chronic hepatitis C prevalence from 1990 to 2023.** Panels show (A) younger than 5 years, (B) 5–9 years, (C) 10–14 years, and (D) 15–19 years. Lines are GBD estimates and shaded bands are supplied 95% uncertainty intervals. GBD, Global Burden of Disease. **Figure S2. Paediatric HCV-related liver cancer burden.** Panels show (A) global prevalence from 1990 to 2023, (B) country and territory prevalence in 2023, (C) global mortality rate from 1990 to 2023, and (D) global DALY rate from 1990 to 2023. Lines indicate GBD estimates and shaded bands indicate supplied 95% uncertainty intervals. Rates are per 100,000 population aged 10–19 years. DALY, disability-adjusted life year; GBD, Global Burden of Disease; HCV, hepatitis C virus. **Figure S3. Relation between 2023 sociodemographic index and paediatric chronic hepatitis C burden among 204 GBD countries and territories.** Vertically arranged panels show (A) prevalence, (B) mortality rate, and (C) DALY rate on linear axes. Each point represents one country or territory and colours indicate the official GBD SDI group. DALY, disability-adjusted life year; GBD, Global Burden of Disease; SDI, sociodemographic index.
