# Supplementary material for: Paediatric chronic hepatitis C and the global elimination gap: burden and trends from 1990 to 2023 in the Global Burden of Disease Study 2023
Source: eClinicalMedicine. 2026 Oct 24;101:104281. doi: 10.1016/j.eclinm.2026.104281 (PMC13720159; doi:10.1016/j.eclinm.2026.104281)
Supplement: Supplementary file 1 [file mmc1.docx]

**Supplementary Materials**

**Supplementary Tables**

Please see larger Excel file for supplementary tables.

**Supplementary Figures**

**Figure S1**

**Figure S2**

**Figure S3**
